# Cardiology hospital admission risk prediction: training, internal validation and technical implementation in the electronic health record

**DOI:** 10.1093/ehjdh/ztag109

**Published:** 2026-07-06

**Authors:** Jasper L Selder, Olivier V Witteman, Oscar M van der Meer, Machteld J Boonstra, Stephan van der Zwaard, Folkert W Asselbergs, Marlies P Schijven, Albert C van Rossum, Cornelis P Allaart

**Affiliations:** Department of Cardiology, Amsterdam Cardiovascular Sciences, Amsterdam University Medical Centre, University of Amsterdam, De Boelelaan 1118, 1081 HZ Amsterdam, The Netherlands; Department of Cardiology, Amsterdam Cardiovascular Sciences, Amsterdam University Medical Centre, University of Amsterdam, De Boelelaan 1118, 1081 HZ Amsterdam, The Netherlands; Department of ICT, Amsterdam University Medical Centre, University of Amsterdam, Amsterdam, The Netherlands; Department of Cardiology, Amsterdam Cardiovascular Sciences, Amsterdam University Medical Centre, University of Amsterdam, De Boelelaan 1118, 1081 HZ Amsterdam, The Netherlands; Department of Cardiology, Amsterdam Cardiovascular Sciences, Amsterdam University Medical Centre, University of Amsterdam, De Boelelaan 1118, 1081 HZ Amsterdam, The Netherlands; Department of Cardiology, Amsterdam Cardiovascular Sciences, Amsterdam University Medical Centre, University of Amsterdam, De Boelelaan 1118, 1081 HZ Amsterdam, The Netherlands; The National Institute for Health Research University College London Hospitals Biomedical Research Centre, University College London, London, United Kingdom; Institute of Health Informatics, University College London, London, United Kingdom; Department of Surgery, Amsterdam UMC, Location University of Amsterdam, Amsterdam, The Netherlands; Amsterdam Gastroenterology and Metabolism, Amsterdam UMC, University of Amsterdam, Amsterdam, The Netherlands; Amsterdam Public Health, Digital Health, Amsterdam UMC, Amsterdam, The Netherlands; Department of Cardiology, Amsterdam Cardiovascular Sciences, Amsterdam University Medical Centre, University of Amsterdam, De Boelelaan 1118, 1081 HZ Amsterdam, The Netherlands; Department of Cardiology, Amsterdam Cardiovascular Sciences, Amsterdam University Medical Centre, University of Amsterdam, De Boelelaan 1118, 1081 HZ Amsterdam, The Netherlands

**Keywords:** Artificial intelligence (AI), Electronic health record, Machine learning, Hospital admission risk prediction, Cardiology outpatients, Clinical decision support

## Abstract

**Aims:**

Rising healthcare demand is increasingly outpacing available outpatient capacity in cardiology, where follow-up is often scheduled at fixed intervals despite substantial variation in individual patient risk. This uniform follow-up approach contributes to high outpatient workload and inefficient use of clinical resources. Accurate risk estimation using routinely collected electronic health record (EHR) data may support more individualized follow-up planning by identifying patients at very low risk of mortality or unplanned hospitalization, in whom follow-up intervals could be safely extended.

**Methods and results:**

We developed and validated a machine-learning model as part of the Cardiology Hospital Admission Risk Prediction (CHARP) program. The retrospective baseline cohort comprised 307 792 outpatient visits from 52 989 unique patients at Amsterdam UMC. The primary endpoint was a composite of unplanned cardiac hospitalization or all-cause death within 2 years; the 1-year composite endpoint served as a secondary outcome. Model development and validation were performed in a filtered, leakage-safe prediction cohort using gradient-boosted decision trees (XGBoost) with strict patient-level GroupKFold cross-validation. All predictions and performance metrics were retrospectively evaluated at the outpatient visit (trigger) level. Retrospective model performance was assessed using AUROC, AUPRC, Brier score, calibration curves, and SHAP-based explainability. The final model was technically deployed within the electronic health record to allow automated, visit-level risk estimation in a prospective silent-running environment. In the filtered prediction cohort (199 961 visits), the 2-year composite endpoint prevalence was 16.8%. Across five cross-validation folds, the CHARP model achieved a mean AUROC of 0.77 ± 0.00 and AUPRC of 0.42 ± 0.01, with a Brier score of 0.12, indicating strong overall discrimination and good calibration. Key predictors included NT-proBNP, renal function indices, prior hospitalizations, and cardiac function measures. The deployed CHARP pipeline successfully generated daily risk predictions for all scheduled cardiology outpatients in the EHR environment throughout the silent-running period.

**Conclusion:**

This study shows that machine-learning applied to routine EHR data can deliver clinically meaningful, visit-level risk stratification for cardiology outpatients. The successful EHR integration of CHARP enables prospective evaluation of data-driven follow-up strategies aimed at reducing outpatient clinic burden through safe de-intensification of follow-up for low-risk patients.

## Graphical Abstract

**Graphical Abstract:**
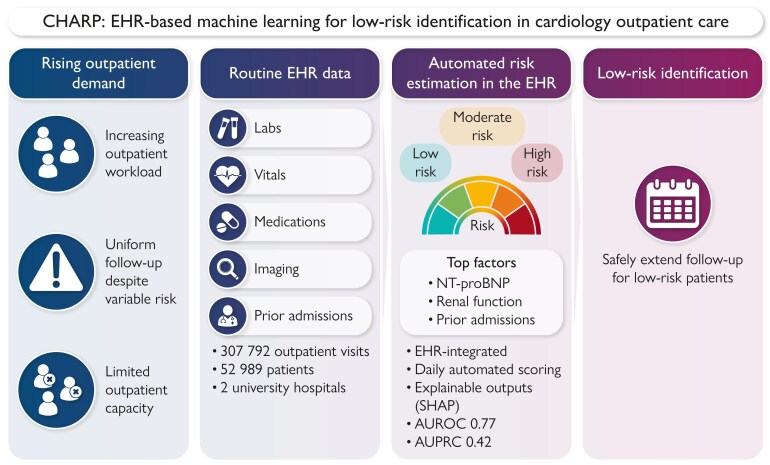

## Introduction

Healthcare demand in Europe is projected to rise substantially over the next 15 years, driven by population growth, aging societies, and changing disease patterns, according to the World Health Organization (WHO).^1^ Although the number of physicians and nurses has increased by ∼10% over the past decade, this growth is unlikely to keep pace with future demand.^1^ Even if workforce targets are to be met, this would be costly, as wages and benefits account for ∼60% of total hospital expenditure.^2^ This makes it imperative to explore innovative strategies to meet rising healthcare needs while using resources efficiently.

A promising approach is the use of Artificial Intelligence (AI) for clinical risk prediction, particularly for unplanned hospital admissions.^3^ Accurate prediction of patients’ future risk may enable clinicians to identify patients at very low risk in whom follow-up intervals can be safely extended or routine outpatient visits reduced, thereby relieving pressure on outpatient capacity. Such an approach focuses on supporting safe de-intensification of routine follow-up while maintaining existing care pathways and clinical oversight. In this way, risk-informed follow-up planning may contribute to more efficient use of healthcare resources without increasing clinical workload.

Although several studies have applied machine learning (ML) to admission or readmission risk prediction, reported performance varies widely and is often difficult to compare due to differences in study populations, outcome definitions, data availability, and methodological limitations such as lack of external validation or potential data leakage.^4–19^ Reported areas under the receiver operating characteristic curve (AUROCs) range from 0.65 to 0.94 across a variety of algorithms, including logistic regression, random forests, gradient boosting, support vector machines, and deep neural networks. While gradient boosting methods frequently achieve strong performance on structured electronic health record (EHR) data, model performance is highly dependent on local care processes, patient case-mix, and data structure. Consequently, transferability across clinical settings is often limited, and models developed in one healthcare system may not generalize to others without local development and evaluation. In particular, machine learning–based prediction of hospital admission risk has not been evaluated in a heterogeneous Dutch tertiary-care cardiology population using routinely collected EHR data within an implementation-oriented framework.

### Aim of the study

In the context of an anticipated mismatch between healthcare demand and available workforce, the overarching program aims to improve the efficiency of cardiology outpatient care through data-driven decision support by identifying patients at very low risk of hospital admission, thereby enabling reduction of unnecessary routine follow-up while maintaining patient safety within existing care pathways. Within this program, the present study specifically aimed to develop, train, and internally validate a machine-learning model (CHARP) to estimate the risk of a composite endpoint of unplanned cardiac hospitalization or all-cause death within 2 years following an outpatient cardiology visit. Secondary objectives were to assess model performance for the same composite endpoint at 1 year and to deploy the algorithm within the hospital EHR to enable silent-running and real-time generation of visit-level risk estimates.

## Methods

### Dataset and data sources

A pseudonymized dataset was constructed from cardiology outpatients of Amsterdam UMC [locations Amsterdam Medical Centre (AMC) and Vrije Universiteit University Medical Centre (VUMC)]. Patients with at least one outpatient cardiology visit between January 2016 and June 2025 were included. Data extraction and harmonization were performed in Snowflake (Snowflake Inc., Bozeman, MT, USA) using curated dynamic views. Clinical variables were standardized across Epic systems using LOINC and SNOMED CT terminology by Medscio (Medscio BV, Amsterdam, The Netherlands). The dataset comprised demographics (age, sex), vital signs, laboratory results, medication exposure, outpatient and inpatient encounters, Diagnosis Treatment Combinations (DBCs), procedures and implantable devices, echocardiography-derived left ventricular ejection fraction, and imaging metadata (modality and timestamp only; no pixel data). Operational definitions of key clinical variables as implemented in Snowflake views are provided in Supplementary material online, *Appendix B*. Each row represented a trigger event, defined as an individual outpatient cardiology visit, serving as the temporal anchor for feature derivation and outcome assessment. Predictors were selected *a priori* based on their availability at or before the outpatient visit, scalability for daily automated deployment in the Epic environment, and robustness against information leakage. We therefore prioritized routinely collected, structured EHR variables that are consistently recorded as discrete fields and harmonized across sites. Variables requiring limited additional processing but with high clinical relevance, such as left ventricular ejection fraction (LVEF), were included using a dedicated extraction workflow. Unstructured data sources (e.g. radiology narratives or free-text reports) were intentionally excluded in this initial version as it would substantially increase pipeline complexity, governance requirements, and leakage risk in a real-time deployment setting.

### Outcome definition

The target variable (TARGET_EVENT) indicated whether unplanned cardiac hospitalization or death occurred after the trigger event. Primary endpoint: Composite of unplanned cardiac hospitalization and all-cause death within 2 years after the trigger date. Secondary endpoint: same composite within 1 year.

### Unit of analysis

The unit of analysis was the trigger level, defined as an individual outpatient cardiology visit. Patients could contribute multiple triggers over time. All analyses were performed at the trigger level, with performance metrics reflecting visit-level risk prediction rather than patient-level outcomes. Trigger visits included all outpatient visits involving a cardiologist or cardiology resident within the Amsterdam UMC cardiology outpatient clinics (AMC and VUMC). This included a broad spectrum of both general and subspecialty cardiology patients, ranging from relatively common outpatient conditions such as coronary artery disease, valvular disease, arrhythmias, and heart failure to more specialized clinics. Visits conducted exclusively by heart failure nurse specialists or atrial fibrillation nurse specialists were not included. Visits outside the cardiology outpatient clinics were also not included. Measures to prevent information leakage, including patient-level data separation and handling of temporally clustered visits, are described in detail in the *Leakage Prevention* section.

### Hospitalization

Hospital admissions were defined as all unplanned admissions to one of the following wards: AMC cardiology ward; AMC coronary care unit; AMC acute cardiac emergency unit; VUMC coronary care unit; VUMC acute cardiac emergency unit; VUMC cardiology ward; AMC cardiothoracic surgery ward; AMC adult intensive care unit (ICU); and VUMC adult intensive care unit (ICU). Admissions were classified as cardiac based on ward type and treating specialty, in accordance with institutional clinical practice. Unplanned admissions were prospectively recorded by nursing staff using a standardized admission-type field within the EHR, in which admissions were coded as planned or unplanned as part of routine clinical workflow.

### Mortality

Deaths were identified through linkage with municipal registries (Statistics Netherlands, CBS) and were labelled as positive if all-cause mortality occurred within the predefined prediction horizon of 2 years (primary objective) and 1 year (secondary objective).

### Leakage prevention

To prevent information leakage and ensure unbiased performance estimation, multiple safeguards were implemented throughout data preprocessing and model development:

- Exclusion of near-event triggers: Outpatient visits occurring within 24 h prior to an unplanned hospital admission were entirely excluded, as these visits are likely to reflect imminent admission-related diagnostics or administrative processes rather than true outpatient decision points.
- Outcome masking: All outcome-related variables (e.g. EVENT_* flags, admission indicators, mortality labels) were removed from the feature set prior to model training.
- Strict temporal anchoring: All predictors were derived using only information available at or before the trigger date. Raw timestamp variables (*_DATE) were used solely for feature derivation and subsequently removed from the final feature matrix to prevent implicit temporal leakage.
- Follow-up requirement and outcome completeness: Triggers with an observed event were retained regardless of follow-up duration, as event occurrence within the 2-year prediction horizon can be ascertained with certainty. For non-event triggers, a minimum follow-up of 550 days was required. This threshold, rather than the full 730 days, was chosen as a pragmatic compromise to avoid unnecessary exclusion of recent, predominantly event-free outpatient visits while limiting false-negative outcome labelling due to incomplete follow-up. Additional sensitivity analyses requiring full 730-day follow-up were performed.
- Near-duplicate trigger deduplication: To reduce redundancy and burst effects, near-duplicate outpatient triggers occurring within 90 days for the same patient were deduplicated in validation and test sets.
- Patient-level data separation: All cross-validation and holdout splits were performed at the patient level, ensuring that data from the same patient never appeared simultaneously in training and validation sets.
- Consistent preprocessing: All preprocessing steps were implemented within a single version-controlled pipeline and applied identically across all validation scenarios.

### Feature engineering

Feature derivation and engineering were implemented in a custom Python pipeline (PipelineCHARPPlus).

- Healthcare and medication utilization:
  - UTIL_INDEX: aggregated exponential-decay index of prior encounters and procedures, w = e^−*λ*⋅Δ^*^t^*, *λ* = 0.01 per day.
  - UTIL_COUNT features: counts of outpatient visits, admissions, and telephone contacts in the preceding 30, 90, and 180 days.
  - Total number of active diagnosis–treatment combinations (DBCs), the Dutch reimbursement unit representing an episode of specialist care for a specific diagnosis, and the number of newly opened DBCs in the preceding 14 months.
  - Medication counts: number of concurrent medications at the trigger date and cumulative prescriptions in the prior 14 months.
- Laboratory results:
  - LV (last value) and BLV (before-last value) prior to the trigger date.
  - Delta features (Δ = LV – BLV).
  - Min/max values for selected biomarkers (e.g. maximum NT-proBNP, maximum troponin).
  - Missing values were kept unchanged, allowing the model to use whether or not a test was performed as potentially informative.
- Vital signs: last and before-last values for blood pressure, heart rate, and BMI, with corresponding delta features.
- Renal function: Chronic Kidney Disease (CKD) stage derived from most recent eGFR, with an additional time-weighted score (CKD_SCORE_TIMEWEIGHTED, *λ* = 0.002/day).
- Cardiac function: LVEF values were extracted using a dedicated semi-automated pipeline combining custom parsing of echocardiography PDF reports with structured retrieval from the GE EchoPAC database (EchoPAC, GE HealthCare, Chicago, Illinois, USA). Successfully extracted values were included in the modelling pipeline, while missing values were handled natively by XGBoost. Extraction accuracy was manually verified in a random subset of patients. Missing values largely reflected heterogeneous report structures in earlier years and the semi-automated nature of the extraction workflow, rather than absence of echocardiographic assessment itself. Procedures and imaging: counts of echocardiograms, angiographies, and other structured procedures.

### Model development

Gradient boosting with XGBoost (binary logistic objective) was used for risk prediction. XGBoost was selected because of its strong empirical performance on large structured EHR datasets, its ability to model non-linear relationships and feature interactions, its native handling of missing data without requiring explicit imputation (although this does not resolve the reasons for missingness). All feature derivation and aggregation were completed upstream in Snowflake, after which the finalized feature matrix was exported for model training and evaluation in Python. Hyperparameters were optimized using Optuna, with the harmonic mean of AUROC and AUPRC as the optimization objective. A representative configuration included a learning rate of ∼0.028, maximum tree depth of 10, minimum child weight of 6, subsampling rate of ∼0.787, column subsampling rate of ∼0.551, L2 regularization (λ) of ∼0.492, L1 regularization (α) of ∼8.541 and up to 1000 boosting rounds with early stopping after 50 rounds. The overall modelling workflow is summarized in Supplementary material online, *Appendix A*.

### Missing value handling

XGBoost’s native missing value handling was used. During tree construction, missing values are automatically directed to the branch that optimizes the split criterion. No external imputation was performed.

### Evaluation

Cross-validation: Model performance was evaluated using five-fold GroupKFold cross-validation with strict patient-level separation. Out-of-fold predictions were used for performance assessment and subgroup analyses. Discrimination was quantified using AUROC and AUPRC (mean ± SD across folds), along with sensitivity, specificity, and precision across probability thresholds. Calibration was assessed on out-of-fold predictions using calibration plots and Brier score; isotonic regression was applied for calibration assessment only and not used to modify model predictions.

Explainability: Model-level: XGBoost’s native importance metrics, gain, weight, and total gain, were extracted across folds, averaged, and visualized for the top 30 predictors.

Trigger-level: SHAP (Shapley Additive Explanations) values were computed for each individual trigger to identify the most influential features contributing to the patient’s risk score. In the EHR, the five most important SHAP-ranked features were displayed alongside the prediction.

### Generalizability and cross-site validation

We evaluated cross-site model transportability using four leakage-free validation scenarios across the two Amsterdam UMC hospital sites (AMC and VUMC), which historically operated as semi-independent tertiary care environments with separate EHR systems for most of the study period. Two scenarios involved full site-exclusive validation, where the model was trained on one site and tested on the other. In addition, we performed patient-exclusive 15% internal holdout validations for each site (ALL → VUMC and ALL → AMC), ensuring that no patient contributed data to both training and testing. The same leakage-prevention procedures used in the main pipeline, including patient-level exclusivity and burst-deduplication of temporally clustered encounters, were applied identically in all cross-site validation scenarios. Although these analyses do not constitute formal external validation, they provide an intermediate assessment of model transportability across distinct clinical environments within a single evolving healthcare system. Model discrimination was assessed using AUROC and AUPRC.

### Model deployment

The trained model was serialized to JSON (JavaScript Object Notation) and deployed in the Amsterdam UMC clinical infrastructure. Execution occurs on a high-performance computing (HPC) cluster positioned between Snowflake (data warehouse) and the Epic EHR.

Each morning, the pipeline runs automatically on all cardiology outpatients scheduled for that day. For every patient, the system generates a probability of unplanned hospitalization or death within 2 years. Alongside the risk score, the five most influential features (SHAP-ranked at trigger level) are returned.

Outputs are integrated in the EHR in silent-running mode and are accessible for research purposes, without being used to guide or influence clinical decision-making. The retrospective analyses presented in this study did not include data from the prospective silent-running deployment. The silent-running setup is intended to facilitate future prospective monitoring of model performance and usability in routine clinical practice, providing the foundation for prospective validation and subsequent randomized evaluation.

### Software and environment

All analyses were conducted in Python (pandas, NumPy, scikit-learn, XGBoost, Optuna, Matplotlib, Seaborn) within Jupyter notebooks. Extensive data preprocessing was executed on Snowflake.

### Risk of bias and applicability

Risk of bias and applicability were evaluated according to the PROBAST + AI framework, covering participant selection, predictors, outcome definition, analysis, and AI-specific leakage domains.

### STROBE and CODE-EHR

This study was reported in accordance with the STROBE statement for cohort studies and the CODE-EHR framework for structured healthcare data. See Supplementary material online, *S1* and *S2*.

## Results

### Study characteristics

#### Baseline characteristics

A total of 52 989 unique patients were included, contributing 307 792 outpatient cardiology visits that served as index triggers for risk prediction. Unless stated otherwise, baseline characteristics are reported at the outpatient visit (trigger) level. The median age at the time of visit was 63 years [interquartile range (IQR) 49–73], and 57% of visits involved male patients. The study population represents a broad outpatient cardiology cohort, with a median body mass index of 25.8 kg/m^2^ (IQR 23.1–29.3), a median heart rate of 70 bpm (IQR 61–80), and a mean arterial pressure of 95 mmHg (IQR 87–104). Hypertension and ischaemic heart disease were present in ∼30% of visits, while 27% involved patients with a history of heart failure and 20% with diabetes mellitus. Renal dysfunction (eGFR <60 mL/min/1.73 m^2^) was observed in 21% of visits, and 3.5% in severe form (eGFR <30 mL/min/1.73 m^2^). Device therapy was common, with 13% of visits involving patients with an implantable cardioverter-defibrillator (ICD) and 2% a CRT-D system. Exact definitions and SQL logic used to derive comorbidity and device flags are provided in Supplementary material online, *Appendix B* (Snowflake definitions).

#### Laboratory and imaging

At the outpatient visit, the median NT-proBNP was 406 ng/L (IQR 130–1310), troponin T 16 ng/L (IQR 8–44), and eGFR 67 mL/min/1.73 m^2^ (IQR 59–86). Median LVEF was 58% (IQR 50–67), among visits with available LVEF data, 11.4% had an LVEF ≤40%.

#### Healthcare utilization and medication

During the preceding 14 months, patients had a mean of 1.9 ± 1.8 hospitalizations and 7.7 ± 8.2 hospital contacts, including 3.7 ± 3.0 outpatient or telephone visits.

Use of medications commonly recommended in cardiovascular guidelines was frequent: 44% of outpatient visits involved β-blockers, 36% ACE inhibitors/ARBs/ARNIs, 16% mineralocorticoid receptor antagonists, and 26% loop diuretics; 3.5% involved SGLT2 inhibitors. Medication exposure was determined through structured medication records up to the outpatient visit, based on substance-level mappings and ATC (Anatomical Therapeutic Chemical)-class groupings.

#### Event rates

Within 2 years after the outpatient visit, the composite endpoint of unplanned cardiac hospitalization or death occurred in 19.1% of visits (14.4% hospitalization, 6.4% death). At 1 year, the composite endpoint occurred in 12.5% (9.9% hospitalization, 3.5% death). These event rates reflect the unfiltered baseline cohort prior to application of model-specific preprocessing and leakage-prevention steps, and are summarized in *Table 1*.

**Table 1:** Baseline characteristics of all cardiology outpatient visits included in the initial analytic cohort before model-specific preprocessing steps

Variable	Overall	2y Event: Yes	2y Event: No	Per patient: first visit	Per patient: last visit	Missing %	Unit/Definition
*n* unique patients	52 989					0.0	Distinct pts
*n* outpatient visits	307 792					0.0	All rows
Baseline characteristics							
Age	63.0 [49.0–73.0]	69.0 [57.0–78.0]	61.0 [47.0–72.0]	59.0 [44.0–71.0]	62.0 [46.0–74.0]	0.0	years
Sex: male	175 440 (57.0%)	33 979 (58.5%)	141 461 (56.7%)	29 224 (55.2%)	29 224 (55.2%)	0.0	*n* (%)
Body mass index	25.8 [23.1–29.3]	26.1 [23.3–29.8]	25.8 [23.1–29.2]	25.5 [22.8–28.9]	25.5 [22.8–28.9]	17.1	kg/m^2^
Heart rate	70.0 [61.0–80.0]	71.0 [62.0–81.0]	70.0 [61.0–80.0]	71.0 [62.0–82.0]	71.0 [62.0–81.0]	12.8	bpm
Mean arterial pressure	95.3 [86.7–104.3]	93.7 [84.3–103.0]	95.7 [87.3–104.7]	95.3 [86.7–104.7]	95.7 [87.0–104.7]	12.3	mmHg
Key risk factors							
Hypertension label	92 426 (30.0%)	23 413 (40.3%)	69 013 (27.6%)	7282 (13.7%)	13 922 (26.3%)	N/a	*n* (%)
Heart failure	81 648 (26.5%)	27 640 (47.6%)	54 008 (21.6%)	6573 (12.4%)	10 600 (20.0%)	N/a	*n* (%)
Diabetes mellitus	62 392 (20.3%)	19 120 (32.9%)	43 272 (17.3%)	6581 (12.4%)	9407 (17.8%)	N/a	*n* (%)
Hypercholesterolemia	18 549 (6.0%)	4736 (8.1%)	13 813 (5.5%)	1738 (3.3%)	2580 (4.9%)	N/a	*n* (%)
Ischaemic heart disease	92 252 (30.0%)	24 844 (42.8%)	67 408 (27.0%)	9102 (17.2%)	12 613 (23.8%)	N/a	*n* (%)
CKD <60 mL/min	65 618 (21.3%)	21 649 (37.3%)	43 969 (17.6%)	7143 (13.5%)	9488 (17.9%)	N/a	*n* (%)
CKD <30 mL/min	10 828 (3.5%)	4793 (8.2%)	6035 (2.4%)	1346 (2.5%)	1887 (3.6%)	N/a	*n* (%)
CRT-D	6071 (2.0%)	1820 (3.1%)	4251 (1.7%)	267 (0.5%)	663 (1.3%)	N/a	*n* (%)
ICD	38581 (12.5%)	9954 (17.1%)	28627 (11.5%)	2869 (5.4%)	3645 (6.9%)	N/a	*n* (%)
Laboratory							
NT-proBNP (LV)	406.0 [129.0–1302.0]	866.0 [271.0–2405.0]	311.0 [108.0–993.0]	317.0 [98.0–1247.0]	331.0 [100.0–1337.8]	47.9	ng/L
Troponin T (LV)	16.0 [8.0–44.0]	20.0 [10.0–50.0]	14.0 [7.0–41.0]	17.0 [7.0–58.0]	17.0 [8.0–53.0]	55.9	ng/L
eGFR (LV)	67.0 [59.0–87.0]	60.0 [48.0–81.0]	69.0 [60.0–88.0]	65.0 [60.0–88.0]	72.0 [60.0–90.0]	17.7	mL/min/1.73 m^2^
Creatinine	84.0 [71.0–102.0]	91.0 [74.0–119.0]	83.0 [70.0–99.0]	80.0 [68.0–96.0]	82.0 [69.0–99.0]	16.5	µmol/L
Sodium	140.0 [138.0–141.0]	139.0 [137.0–141.0]	140.0 [138.0–142.0]	140.0 [138.0–142.0]	140.0 [138.0–141.0]	21.4	mmol/L
Potassium	4.2 [4.0–4.5]	4.2 [4.0–4.6]	4.2 [4.0–4.5]	4.2 [3.9–4.4]	4.2 [3.9–4.5]	21.2	mmol/L
CRP	3.1 [1.5–12.0]	4.0 [1.9–13.9]	3.0 [1.4–11.3]	3.6 [1.7–14.0]	3.4 [1.5–14.2]	32.6	mg/L
Haemoglobin	8.5 [7.7–9.2]	8.3 [7.4–9.1]	8.5 [7.8–9.2]	8.5 [7.7–9.2]	8.4 [7.6–9.1]	18.2	mmol/L
Echocardiography							
LVEF	58.0 [50.0–67.0]	53.0 [42.0–64.0]	59.0 [52.0–68.0]	58.0 [51.0–67.0]	58.0 [51.0–67.0]	82.9	%
LVEF ≤40%	5996 (11.4%)	2404 (22.9%)	3592 (8.5%)	1402 (9.0%)	1402 (9.0%)	82.9	*n* (%)
Prior healthcare utilization							
Hosp last 14m	1.9 ± 1.8	2.4 ± 2.3	1.7 ± 1.4	1.4 ± 0.9	1.5 ± 1.2	73.3	*n*
Hosp contacts last 14 m	7.7 ± 8.2	11.0 ± 10.1	6.9 ± 7.4	3.8 ± 6.1	6.1 ± 7.4	0.0	*n*
OPV/calls last 14m	3.7 ± 3.0	4.9 ± 4.0	3.4 ± 2.6	1.1 ± 0.4	2.7 ± 2.1	0.0	*n*
Medication							
ACEi/ARB/ARNI	111 170 (36.1%)	30 806 (53.0%)	80 364 (32.2%)	7019 (13.2%)	15 316 (28.9%)	N/a	*n* (%)
Beta-blocker	135 137 (43.9%)	35 238 (60.6%)	99 899 (40.0%)	8101 (15.3%)	18 622 (35.1%)	N/a	*n* (%)
MRA	49 871 (16.2%)	17 450 (30.0%)	32 421 (13.0%)	2533 (4.8%)	6240 (11.8%)	N/a	*n* (%)
Loop diuretic	81 203 (26.4%)	27 085 (46.6%)	54 118 (21.7%)	5500 (10.4%)	11 077 (20.9%)	N/a	*n* (%)
SGLT2 inhibitor	10 914 (3.5%)	4313 (7.4%)	6601 (2.6%)	486 (0.9%)	2110 (4.0%)	N/a	*n* (%)
Calcium channel blocker	92696 (30.1%)	24495 (42.1%)	68201 (27.3%)	6454 (12.2%)	13591 (25.6%)	N/a	*n* (%)
Thiazide	26 155 (8.5%)	7571 (13.0%)	18 584 (7.4%)	1768 (3.3%)	3728 (7.0%)	N/a	*n* (%)
Event rates							
1-year hospitalization	30 586 (9.9%)						
2-year hospitalization	44 419 (14.4%)						
1-year death	10 783 (3.5%)						
2-year death	19 809 (6.4%)						
1-year composite	38 525 (12.5%)						
2-year composite	58 701 (19.1%)						

This cohort represents the full set of outpatient visits prior to applying follow-up requirements, exclusion of visits occurring <24 h before an unplanned admission, and near-duplicate trigger deduplication used for machine-learning model training and validation. Event rates (1-year and 2-year hospitalization, death, and their composite) reflect unadjusted outcomes in the unfiltered baseline cohort. Subsequent model performance metrics and subgroup analyses are based on the filtered prediction cohort after applying these preprocessing steps. For binary comorbidity and medication flags, missingness is not applicable. Reported 2-year event rates may slightly underestimate true incidence due to incomplete follow-up of recent visits.

### Model performance

#### Discrimination

**Figure 1:**
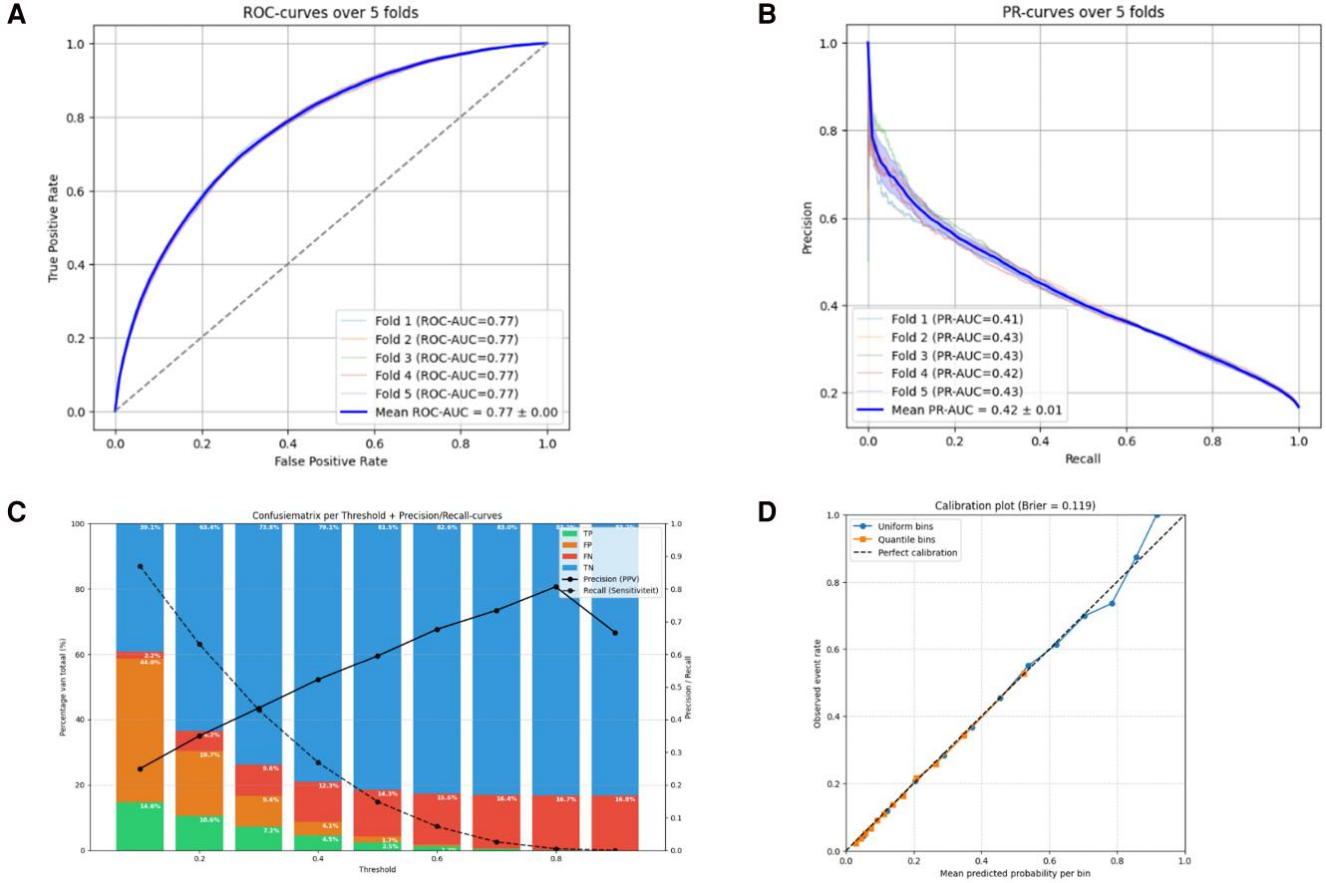
Discrimination and calibration performance of the CHARP model for predicting the 2-year composite endpoint of unplanned cardiac hospitalization or death. (*A*) Receiver operating characteristic (ROC) curves across five GroupKFold validation folds. (*B*) Precision–recall (PR) curves across the same folds. (*C*) Threshold analysis illustrating the trade-off between precision, recall, and class proportions across probability cut-offs. (*D*) Calibration plot showing agreement between predicted and observed event rates, with overall Brier score of 0.119.

For prediction of the composite endpoint within 2 years, model performance was evaluated in the filtered, leakage-safe prediction cohort (*n* = 199 961) after application of all preprocessing and leakage-prevention steps described in the Methods. Across five patient-level GroupKFold validation folds, the mean AUROC was 0.77 ± 0.00 and the AUPRC was 0.42 ± 0.01, given an overall 2-year event prevalence of 16.8%. Discrimination and threshold performance are illustrated in *Figure 1A–C*, displaying the ROC and PR curves together with the precision-recall-specificity profiles across thresholds.

#### Calibration

Calibration analysis showed close agreement between predicted and observed event rates across deciles of predicted risk. The calibration curve closely followed the ideal 45° line, indicating minimal systematic bias; additional isotonic or logistic recalibration was therefore not required during model development and internal validation. The Brier score was 0.119, reflecting good overall accuracy of the probabilistic predictions (*Figure 1D*).

#### Sensitivity analyses

Follow-up completeness sensitivity analysis: Requiring full 730-day follow-up for apparently event-free triggers reduced the prediction cohort from 199 961 to 193 200 outpatient visits, while model performance remained materially unchanged (AUROC 0.769 vs. 0.768; AUPRC 0.419 vs. 0.427; Brier score 0.118 vs. 0.121), supporting robustness of the primary findings to stricter follow-up completeness requirements (see Supplementary material online, *Appendix C*).

Endpoint-specific sensitivity analyses: For 2-year unplanned cardiac hospitalization alone, the model achieved an AUROC of 0.751 (SD 0.004) and an AUPRC of 0.312 (SD 0.014). For 2-year all-cause mortality alone, AUROC was 0.873 (SD 0.004) and AUPRC was 0.335 (SD 0.013). A shorter 1-year composite endpoint yielded similar discrimination performance (AUROC 0.766, SD 0.002; AUPRC 0.309, SD 0.007) compared with the primary 2-year composite endpoint (AUROC 0.769, SD 0.003; AUPRC 0.418, SD 0.006) (see Supplementary material online, *Appendix D*).

Benchmark comparison with logistic regression: Compared with regularized logistic regression using the same feature set and validation strategy, XGBoost achieved superior performance (AUROC 0.77 vs. 0.74; AUPRC 0.42 vs. 0.39; Brier score 0.118 vs. 0.188), supporting the added value of non-linear modelling and feature interactions in structured EHR data.

#### Feature importance and explainability

**Figure 2:**
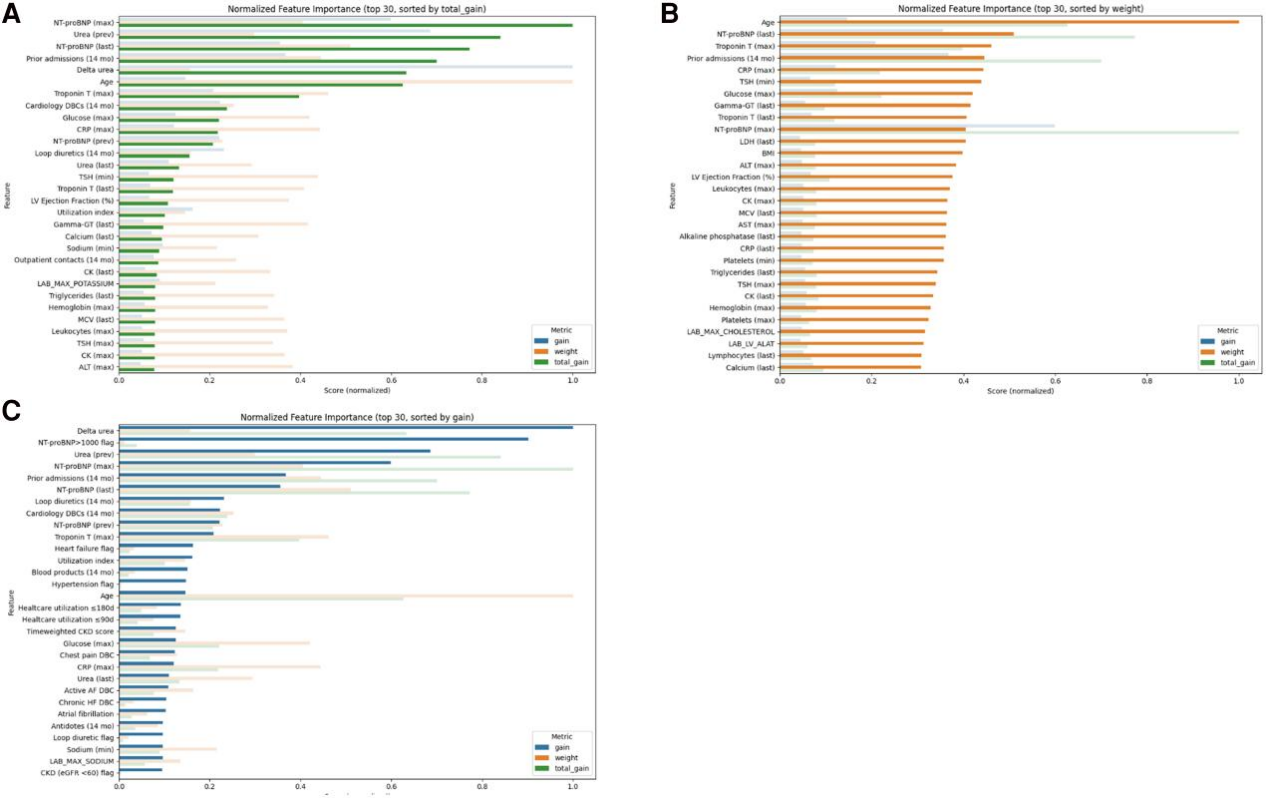
Top 30 model features ranked by three XGBoost importance metrics: (*A*) total gain (cumulative improvement in model performance), (*B*) weight (frequency of use in tree splits), and (*C*) gain (average improvement per split).

Feature importance was evaluated across all validation folds using XGBoost’s intrinsic metrics: Gain, representing the average improvement in model performance (information gain) brought by a feature when used for splitting; Total gain, the sum of all gains for a given feature across all trees; Weight, the number of times a feature was used for splitting within the model. The top 30 features ranked by these metrics are shown in *Figure 2*.

Across all three measures, variables related to NT-proBNP and renal function consistently ranked highest. Maximum NT-proBNP, NT-proBNP>1000 flag, and recent NT-proBNP values were the most influential predictors, followed by serum urea levels (maximum, before-last, and delta values) and the presence of CKD.

**Figure 3:**
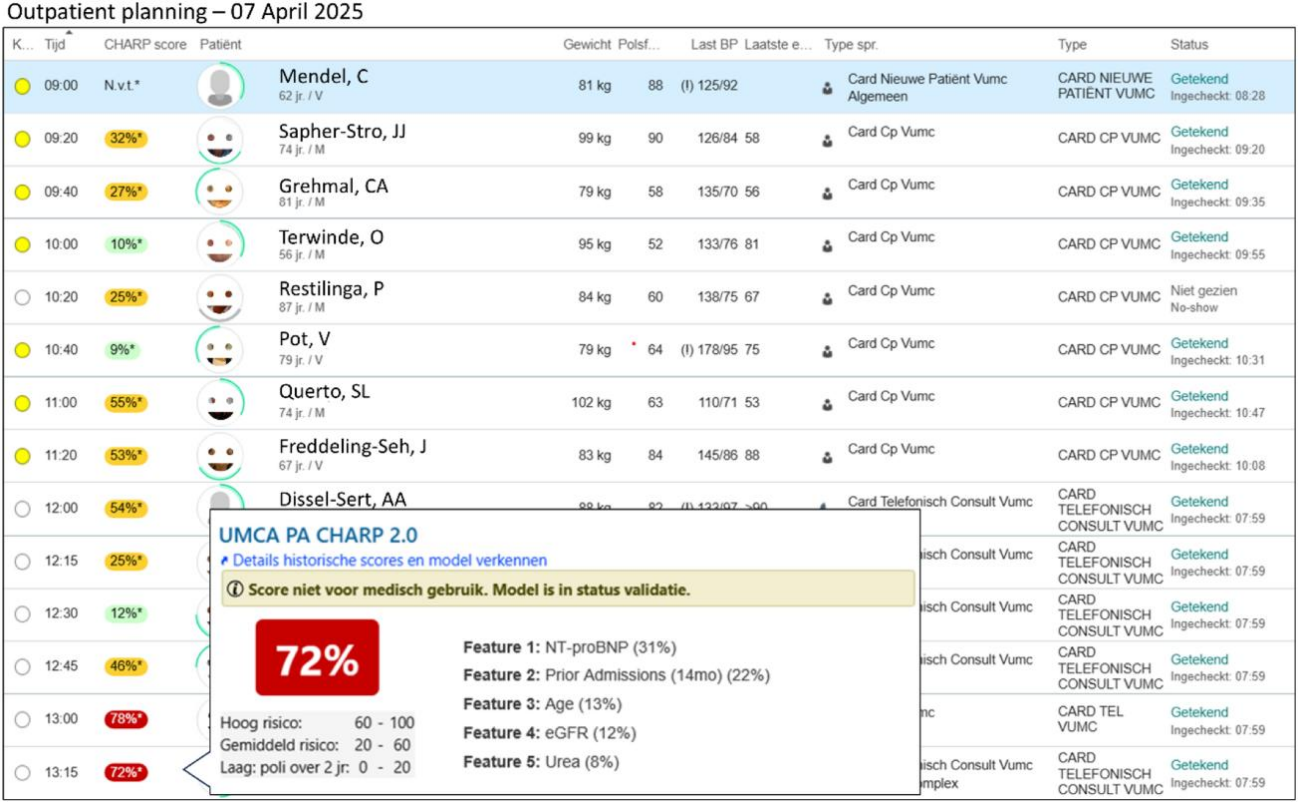
Example of a cardiology specialist’s outpatient agenda displaying a single highlighted visit with a predicted 2-year risk of unplanned cardiac hospitalization or death of 72%, including the corresponding SHAP-based feature contributions for that visit. Feature contributions represent local explanations relative to the model’s expected value. Patient identifiers, including names and ages, have been modified for privacy purposes. At the time of analysis, the model was deployed in silent-running mode, with predictions and explanations visible to researchers only and not used for clinical decision-making. Image adapted for research purposes. Copyright © Amsterdam UMC. Epic® is a registered trademark of Epic Systems Corporation.

Indicators of healthcare utilization, including prior admissions, outpatient contacts, and encounter counts in the preceding months, also contributed substantially to model performance. Additional features with notable importance included troponin T, C-reactive protein, serum glucose, age, and flags for heart failure, hypertension, and reduced LVEF ≤ 40%. Local model explainability was assessed using Shapley Additive Explanations (SHAP) to quantify, for each individual prediction, the direction and magnitude of each feature’s contribution to the predicted risk. For every outpatient visit (‘trigger’), SHAP values were computed to identify which variables most strongly increased or decreased the probability of cardiac hospitalization or death. During model deployment, the five highest-impact features (based on absolute SHAP values) are displayed within the electronic medical record alongside the predicted probability, enabling clinicians to understand why a specific patient is flagged as higher or lower risk. *Figure 3* illustrates an example of an outpatient visit with a predicted 2-year risk of 72%, including the corresponding SHAP-based feature contributions.

#### Subgroup analysis

Model performance was similar across all major clinical subgroups (*Table 2*). Discrimination and calibration metrics showed limited variation between subgroups. AUPRC was higher in subgroups with higher event rates, including CKD and heart failure. Overall performance metrics remained comparable across the evaluated cardiovascular comorbidities.

**Table 2:** Out-of-fold model performance across major clinical subgroups, showing event rates and discrimination metrics (AUROC and AUPRC) together with overall probabilistic accuracy expressed as the brier score

Subgroup	*n* visits	Events	Event rate	AUROC	AUPRC	Brier
**CKD (eGFR<30)**	6430	2674	0.42	0.74	0.64	0.20
**Heart failure**	47249	14752	0.31	0.72	0.53	0.19
**CKD (eGFR<60)**	39522	12074	0.31	0.73	0.54	0.18
**Diabetes**	38013	10774	0.28	0.73	0.52	0.17
**Age ≥70y**	69003	17297	0.25	0.72	0.48	0.16
**Ischaemic heart disease**	56674	14087	0.25	0.73	0.48	0.16
**Hypertension**	54953	12837	0.23	0.74	0.47	0.15
**All patients**	199961	33627	0.17	0.77	0.42	0.12

Visit counts reflect the filtered prediction cohort used for out-of-fold validation and are therefore lower than the unfiltered baseline cohort shown in *Table 1*. Differences in Brier score across subgroups largely reflect differences in event prevalence and should not be interpreted as subgroup-specific miscalibration. Overall model calibration was assessed in the full cohort.

#### Generalizability and cross-site performance

After preprocessing, model performance was evaluated across four validation settings. In the site-exclusive experiments, training on VUMC data and testing on AMC data resulted in an AUROC of 0.737 and an AUPRC of 0.360, while training on AMC data and testing on VUMC data yielded an AUROC of 0.725 and an AUPRC of 0.420. In the patient-exclusive internal evaluations, where 15% of patients per site were reserved as a holdout set, training on the combined dataset excluding 15% of VUMC patients and testing on the VUMC holdout cohort resulted in an AUROC of 0.748 and an AUPRC of 0.456. Similarly, training on the combined dataset excluding 15% of AMC patients and testing on the AMC holdout cohort achieved an AUROC of 0.786 and an AUPRC of 0.417.

#### Model deployment

**Figure 4:**
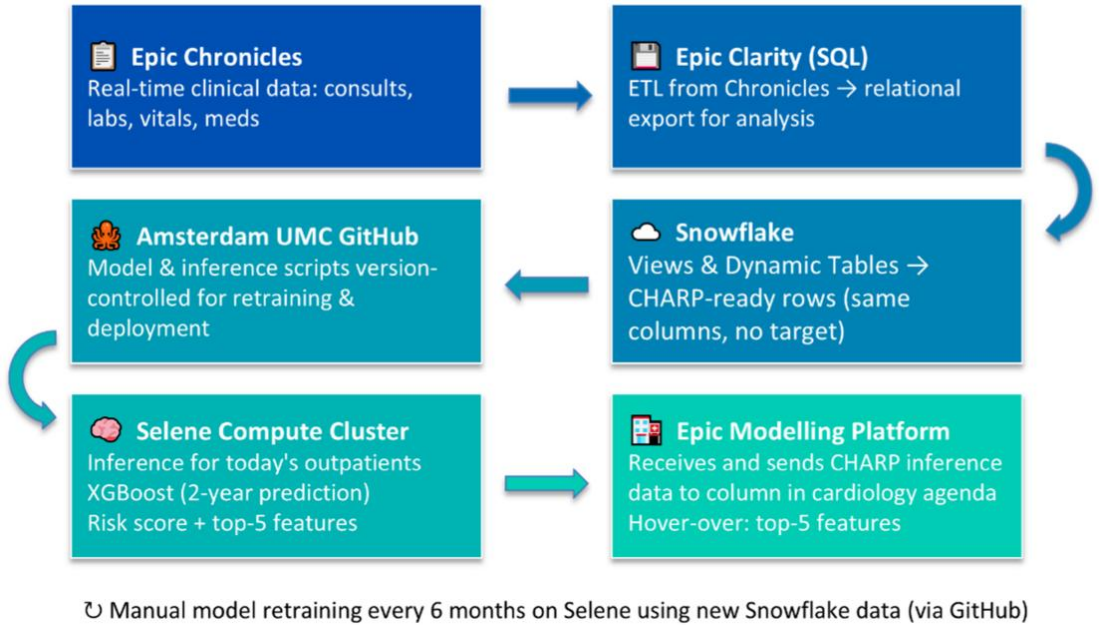
System architecture of the CHARP pipeline, illustrating data flow from real-time clinical sources in epic to inference and deployment across Snowflake, Selene compute cluster, and the epic modelling platform, with planned biannual model retraining managed via GitHub.

The CHARP model was fully integrated within the hospital’s Epic environment and data platform. Clinical data originate in Epic Chronicles, Epic’s non-relational transactional database that underpins the live EHR. For analytical and research use, data are replicated into Epic Clarity, a relational database optimized for reporting, and subsequently exposed through curated Snowflake views representing the outpatient cardiology cohort and derived features used by the model. Each morning at 06:00, all patients scheduled for the outpatient cardiology clinic that day are automatically retrieved from Epic. Their data are transformed using the same feature-engineering logic as in model training and sent to the CHARP inference cluster, a containerized service version-controlled in Git. The resulting 2-year composite risk predictions are returned through Epic’s Cognitive Computing/Nebula predictive-modelling framework and written back into the operational Epic environment, as illustrated in *Figure 4*. Within Epic Hyperspace, the risk score appears as an additional column in the outpatient clinic agenda. A hover-over tooltip displays the five most influential features for each prediction, providing transparent and interpretable decision support. The system runs fully automatically, without manual input, and scores all scheduled patients once daily.

The present study reports retrospective model development and validation results only. Although the model has been technically deployed in a prospective silent-running environment, outputs generated during this phase were not used to guide clinical decision-making and were not included in the current analyses. Prospective silent-running evaluation results will be reported separately.

#### Risk of bias and applicability

Using the updated PROBAST + AI framework,^20^ the CHARP model demonstrated a low overall risk of bias and high applicability to outpatient cardiology. Participants: The cohort consisted of all cardiology outpatients across two academic hospitals within one healthcare system. This ensures internal consistency but may limit generalizability, consistent with a moderate applicability concern due to the single-system setting. Predictors: All predictors were routinely collected EHR variables available at, or before, the outpatient visit. Extensive leakage-prevention measures, removal of raw timestamps, exclusion of <24 h pre-admission data, patient-level splits, and deduplication of near-duplicate visits, yielded a low risk of predictor-related bias, including AI-specific leakage domains introduced in PROBAST + AI (temporal, sampling, and pipeline leakage). Outcome: Outcomes were defined using structured internal hospital codes and municipal mortality linkage. A residual limitation is incomplete capture of unplanned cardiac hospitalizations in external hospitals, leading to possible under-ascertainment of true events. PROBAST + AI classifies this as a potential outcome-misclassification risk. Analysis: The use of patient-level GroupKFold, strict feature-timestamp masking, gradient boosting with robust missing-handling, and calibration assessment aligns with PROBAST + AI’s recommendations for AI-based prediction models. Overall analysis-related bias was low, with adequate sample size, transparent hyperparameter optimization, and reproducible pipelines. Applicability: Applicability to similar EHR-rich cardiology settings is high; external validation in independent systems will be required to mitigate the moderate applicability concern arising from single-system derivation.

## Discussion

In this large-scale study of over 300 000 outpatient cardiology visits across two academic centres, we developed and validated a machine learning (ML) model, CHARP, to predict unplanned cardiac hospitalization or death within two years after an outpatient visit. The model achieved robust discrimination (AUROC 0.77; AUPRC 0.42) and good calibration (Brier 0.12), while maintaining consistent performance across clinically relevant subgroups and across the two historically distinct Amsterdam UMC hospital sites, supporting transportability across different clinical environments within the same healthcare system. These findings show that routinely collected EHR data can be used to estimate individualized risk for adverse cardiac outcomes and may support identification of patients at very low risk in whom routine follow-up intensity could be reduced.

### Comparison with prior studies

Previous machine learning (ML) approaches to hospitalization or readmission risk prediction have typically shown modest performance (AUROC 0.65–0.75) and limited transportability beyond their derivation cohorts.^4–14^ However, many existing clinical models focus specifically on short-term readmission risk, typically predicting 30-day rehospitalization following hospital discharge, rather than longer-term risk of unplanned hospitalization and death from the outpatient setting. As such, direct comparison with these models is inherently limited due to differences in prediction target, clinical context, and time horizon. CHARP’s discriminative ability nevertheless compares favourably with these reports and with contemporary readmission models used in clinical operations, such as the Epic Readmission Risk Model and the LACE index, which generally perform in the 0.65–0.70 range and are designed to estimate short-term readmission risk rather than longer-term hospitalization risk.^18^ In contrast, CHARP predicts the risk of unplanned cardiac hospitalization or death over a 2-year time horizon, reflecting a different clinical use case focused on longitudinal outpatient risk stratification. The gain in accuracy likely reflects the comprehensive feature engineering strategy, the use of gradient boosting (XGBoost) with extensive hyperparameter optimization, and rigorous prevention of data leakage at both feature and patient levels. Importantly, model calibration, often neglected in high-dimensional ML studies, was good in the current internal validation setting without the need for immediate post-hoc recalibration, supporting clinical interpretability and safe use for probabilistic risk estimation.

### Key predictors and pathophysiological insights

The strongest predictors of cardiac hospitalization and death were NT-proBNP, markers of renal function (eGFR, urea, creatinine), and prior healthcare utilization. This aligns with known cardio-renal interactions, where elevated natriuretic peptides and reduced renal function jointly indicate haemodynamic stress, volume overload, and systemic vulnerability. Other influential variables included troponin T, C-reactive protein, age, and prior heart failure, all consistent with established prognostic determinants in chronic cardiovascular disease. Notably, sex did not emerge as a major independent predictor. Global predictor importance was primarily derived from native XGBoost importance metrics, while SHAP values were additionally used to support visit-level explainability within the EHR interface. Importantly, both approaches identified broadly similar dominant predictors. The model’s explainability displayed directly in the electronic medical record, allows clinicians to understand these relationships at the individual-patient level, which may foster trust and facilitate adoption.^21^

### Clinical and operational implications

The CHARP program aims to support risk-informed outpatient care by identifying patients at low risk for hospital admission or death in whom follow-up intervals may be safely extended, thereby reducing unnecessary routine visits while maintaining existing care pathways and clinical oversight. Predictions reflect observed outcomes under current care pathways rather than underlying untreated risk. Therefore, in individual patients, treating physicians may decide that intensified follow-up itself contributes to clinical stability and should therefore be maintained. At the same time, one of the central hypotheses underlying the CHARP program is that a substantial proportion of routine outpatient follow-up visits in current practice may not materially alter short- to medium-term outcomes. Prospective evaluation will therefore be necessary to determine whether model-informed follow-up de-intensification can be implemented safely without unintended effects on patient outcomes.

When validated prospectively, such risk-informed follow-up strategies could alleviate pressure on outpatient capacity without compromising safety. Given that outpatient cardiology visits represent one of the highest-volume services in European tertiary care, even modest efficiency gains could translate into substantial system-level impact.

Although the present study reports retrospective model development and internal validation only, the model has already been technically deployed in a prospective silent-running environment fully integrated within the Epic EHR. In this setting, predictions are generated automatically for all scheduled outpatients but are not yet visible and for clinicians and not yet used to support clinical decision-making. This infrastructure enables future prospective evaluation of real-world model behaviour, calibration drift, and clinician interaction prior to implementation of live decision support. Importantly, real-world EHR integration and deployment infrastructure remain insufficiently described in the current prediction-model literature, despite being critical prerequisites for prospective clinical evaluation and implementation. The overall architecture therefore reflects a learning health-system framework in which model performance, data quality, and outcomes can be continuously monitored and evaluated.

### Strengths and innovations

This study combines several methodological and translational strengths, including a harmonized longitudinal EHR infrastructure across two historically distinct hospital sites within one academic healthcare system using standardized SNOMED-CT and LOINC terminology; a feature-engineering framework capturing healthcare utilization, biomarker trajectories, and temporal clinical patterns; extensive safeguards against temporal and patient-level leakage, including strict patient-level separation and near-duplicate visit handling during validation; full integration into the operational EHR infrastructure for automated daily visit-level risk estimation; trigger-level explainability within the EHR using SHAP-based feature attribution; and an implementation-oriented design focused on identifying patients at very low risk to support safe de-intensification of routine outpatient follow-up. Together, these features position CHARP as a scalable and implementation-ready framework for prospective evaluation of AI-supported outpatient care optimization.

### Limitations

Several limitations warrant consideration. First, although the dataset was comprehensively harmonized, it originates from a single evolving healthcare system encompassing two historically distinct tertiary hospital sites. Therefore, true external validation in independent healthcare systems remains necessary to assess broader transportability and clinical applicability. However, for complex, high-dimensional EHR-based prediction models such as CHARP, strict cross-institution generalizability may not always be the primary objective. Local use and retraining or recalibration in external hospitals may yield superior discrimination by capturing institution-specific clinical practices, case mix, and data-generation processes.^16^ This raises an important conceptual trade-off between global generalizability and locally optimized predictive performance, which should be explicitly addressed in future deployment studies.

Second, although the endpoint already included a broad range of acute cardiac and critical care departments, certain clinically related non-cardiac admissions may still have been missed, particularly when managed without cardiology involvement or when occurring at hospitals outside Amsterdam UMC.

Third, the model relies exclusively on structured EHR variables. Unstructured clinical notes, raw imaging data, and genomic information were not incorporated. While this design choice enhances transparency and facilitates deployment, it likely constrains the upper bound of predictive performance. Future multimodal extensions integrating natural language features and imaging-derived biomarkers may further improve discrimination and clinical relevance.

Fourth, although XGBoost natively accommodates missing values and may leverage predictive information contained in missingness patterns, this does not resolve the reasons why values are missing, particularly when data are missing not at random. In EHR-derived datasets, missingness itself may encode clinically meaningful or operational information, such as selective laboratory ordering or workflow-related data availability. These patterns may also vary across institutions and could affect model transportability if not explicitly evaluated or recalibrated. Furthermore, although XGBoost provides strong predictive flexibility, more interpretable models such as logistic regression may be preferable when mechanistic interpretation or targeted interventions are the primary objective. Fifth, the current implementation operates in silent-running mode. Consequently, the impact of CHARP on clinician behaviour, workload, decision-making, and patient outcomes has not yet been evaluated. Prospective studies, including randomized or stepped-wedge designs, will be necessary to assess real-world clinical effectiveness, safety, and unintended consequences prior to routine clinical use.

Sixth, because the primary endpoint was designed to reflect overall cardiology outpatient vulnerability and healthcare demand rather than a single disease-specific cardiovascular process, CHARP should primarily be interpreted as a pragmatic outpatient risk-stratification tool for follow-up optimization rather than a disease-specific prognostic model.

Seventh, although strict patient-level separation and 90-day near-duplicate visit deduplication were applied during validation, residual clustering at provider-, clinic-, or hospital-level may still persist.

Finally, deployment of CHARP may influence clinical behaviour, potentially altering predictor distributions and event incidence over time. This may result in calibration drift despite stable discrimination, highlighting the need for prospective monitoring and periodic recalibration during implementation.

### Future directions

The next phases of the CHARP program will follow a staged translational roadmap. First, prospective silent-running validation will be conducted to assess real-time model performance, calibration drift, and temporal robustness in routine clinical care. Second, CHARP will be implemented as a decision support tool within the clinical workflow, explicitly designed to support, rather than replace, clinical judgment. This phase will focus on safe integration with full clinician override, evaluation of usability and interpretability, and assessment of potential behavioural effects on clinical decision-making. Third, a randomized clinical trial will evaluate the clinical impact of CHARP-guided care, including effects on outpatient visit frequency, unplanned hospitalizations and mortality. In parallel, existing explainability dashboards and user-interface components, already implemented within the current CHARP infrastructure, will be iteratively optimized in close collaboration with clinicians to further enhance transparency, usability, and trust. Equally important, future work will focus on establishing robust model governance and lifecycle management, including version control, performance monitoring, recalibration strategies, and clearly defined ownership of long-term maintenance, which are essential for safe and sustainable deployment in routine care. Lastly, model improvement will be achieved through systematic data enrichment through accumulation of additional outpatient data and inclusion of newly available variables. Periodic retraining (e.g. every year) will account for temporal changes in case-mix and clinical practice, while incorporation of longitudinal biomarker trajectories, healthcare utilization dynamics, and structured imaging-derived features may improve discrimination and calibration. All updates will be evaluated using strict temporal holdout testing within the same leakage-prevention framework to ensure real-world validity. Beyond the specific CHARP implementation, similar AI-supported risk stratification approaches may also be applicable to other clinical settings, such as post-emergency department follow-up pathways and additional outpatient specialties where routine follow-up schedules may contribute to unnecessary healthcare utilization.

### Conclusion

The CHARP model provides a scalable, fully EHR-integrated approach for visit-level risk estimation in outpatient cardiology. The model demonstrated good discrimination, enabling identification of patients at very low risk, and allows prospective evaluation of risk-informed follow-up strategies aimed at safely reducing routine follow-up intensity while maintaining quality and safety of care.

## Supplementary Material

ztag109_Supplementary_Data

## Data Availability

The data underlying this article cannot be shared publicly due to patient privacy regulations, institutional data governance policies, and the use of protected electronic medical record data. De-identified data may be shared on reasonable request to the corresponding author, subject to approval by Amsterdam UMC and applicable legal and institutional governance procedures.
