# Supplementary material for: Cardiology hospital admission risk prediction: training, internal validation and technical implementation in the electronic health record
Source: Eur Heart J Digit Health. 2026 Jul 6;7(7):ztag109. doi: 10.1093/ehjdh/ztag109 (PMC13419068; doi:10.1093/ehjdh/ztag109)
Supplement: ztag109_Supplementary_Data [file ztag109_supplementary_data.zip › Appendix C and D.docx]

# Appendix C. Sensitivity analysis evaluating stricter minimum follow-up duration requirements for apparently event-free outpatient visits

| **Scenario** | **N** | **Events (%)** | **AUROC** | **AUPRC** | **Brier** |
| --- | --- | --- | --- | --- | --- |
| ≥550-day follow-up for non-events (main analysis) | 199,961 | 33,627 (16.8%) | 0.769 ± 0.003 | 0.419 ± 0.006 | 0.118 |
| ≥730-day follow-up for non-events (sensitivity analysis) | 193,200 | 33,627 (17.4%) | 0.768 ± 0.003 | 0.427 ± 0.006 | 0.121 |

# Appendix D. Endpoint-specific sensitivity analyses

| **Endpoint** | **AUROC (SD)** | **AUPRC (SD)** | **Brier score** |
| --- | --- | --- | --- |
| 2-year composite | 0.769 (0.003) | 0.418 (0.006) | 0.118 |
| 2-year hospitalization only | 0.751 (0.004) | 0.312 (0.014) | 0.097 |
| 2-year mortality only | 0.873 (0.004) | 0.335 (0.013) | 0.045 |
| 1-year composite | 0.766 (0.002) | 0.309 (0.007) | 0.084 |
