# Supplementary material for: Cardiology hospital admission risk prediction: training, internal validation and technical implementation in the electronic health record
Source: Eur Heart J Digit Health. 2026 Jul 6;7(7):ztag109. doi: 10.1093/ehjdh/ztag109 (PMC13419068; doi:10.1093/ehjdh/ztag109)
Supplement: ztag109_Supplementary_Data [file ztag109_supplementary_data.zip › Supplementary file.docx]

# Appendix A. Workflow of the CHARP Prediction Pipeline

Overview of data flow, preprocessing, model development, evaluation, and deployment.

## 1. Data extraction and harmonization

Clinical data were extracted from curated Snowflake views representing the outpatient cardiology population. Extracted domains included demographics, vital signs, laboratory results, medications, procedures, encounters, and imaging metadata (modality and timestamp only; no pixel data). Variables were harmonized across Epic systems using standardized LOINC and SNOMED CT mappings.

## 2. Preprocessing and leakage prevention

To prevent information leakage, several safeguards were applied prior to model training:

• Masking and exclusion of data occurring ≤24 hours before an unplanned admission.

• Deduplication of near-duplicate outpatient triggers occurring within 90 days.

• Removal of all raw timestamp variables (*_DATE) after feature derivation.

## 3. Feature engineering

Derived features captured both clinical status and longitudinal care patterns:

• Healthcare utilization: exponential-decay utilization index (UTIL_INDEX), encounter counts over 30/90/180 days (UTIL_COUNT), Diagnosis Treatment Combination (DBC) counts, and medication counts.

• Laboratory and vital signs: last value (LV), before-last value (BLV), delta (LV–BLV), and selected minimum/maximum values.

• Renal function: chronic kidney disease (CKD) stage and a time-weighted CKD score (CKD_SCORE_TIMEWEIGHTED).

• Cardiac function: left ventricular ejection fraction (LVEF) extracted using a custom script from echocardiography reports and vendor databases.

• Procedures and imaging: counts of echocardiography, coronary angiography, and other structured procedures.

## 4. Model development

Risk prediction was performed using gradient boosting (XGBoost) with native handling of missing values. Hyperparameters were optimized using Optuna. Model development and internal validation employed patient-level GroupKFold cross-validation to ensure complete separation of individuals across folds.

## 5. Model evaluation and explainability

Performance was assessed using AUROC, AUPRC, calibration plots, and Brier score.

Explainability was implemented at two levels:

• Trigger-level: SHAP values identifying the five most influential features for each individual prediction.

• Model-level: XGBoost feature importance metrics (gain, weight, and total gain).

## Deployment (silent run)

The trained model was deployed on a high-performance computing cluster positioned between Snowflake and the Epic EHR. The pipeline runs daily and generates 2-year risk estimates for all scheduled outpatient cardiology visits. During the silent-running phase, predictions and SHAP explanations are displayed in the EHR for monitoring and validation purposes only and are not used to guide clinical decision-making.

# Appendix B. Definitions of Key Clinical Variables in Snowflake

### Hypertension

Hypertension (HT_FLAG) was defined using three independent electronic health record signals:

1. Diagnosis codes: hypertension-related Diagnosis Treatment Combinations (DBCs: 0320.902, 0313.311, 0316.4003, 8416.0107, 8416.0106, 0318.901), excluding pulmonary and ocular hypertension.
2. Blood pressure: mean arterial pressure (MAP; VIT_LV_BLOOD_PRESSURE) ≥110 mmHg, corresponding approximately to systolic/diastolic values of 150/100 mmHg.

This threshold was selected to reduce misclassification due to transient hospital-related blood pressure elevation.

1. Medication: antihypertensive therapy weighted by class: thiazides, calcium channel blockers, and α1-blockers (1 point); RAAS inhibitors and β-blockers (0.5 points); loop diuretics (0 points). A cumulative score ≥2 classified treated hypertension.

Patients were classified as hypertensive if any of these criteria were met.

### Heart Failure

Heart failure (HF_FLAG) was defined using a composite of diagnostic, functional, biomarker, device, and medication-based signals:

1. Diagnosis codes: heart failure–related DBCs (e.g., 0320.301, 0320.302, 0320.303, 0320.305, 0320.306, 8416.0105).
2. Cardiac function: LVEF <40% on echocardiography or cardiac MRI.
3. Biomarker: NT-proBNP >1000 ng/L.
4. Device therapy: presence of an ICD or CRT-D (implantation, replacement, or follow-up).
5. Medication: guideline-directed heart failure therapy (RAAS inhibitor/ARNI, β-blocker, MRA, loop diuretic, SGLT2 inhibitor). Patients using ≥4 drug classes, or an ICD plus ≥3 classes, were classified as having heart failure.

Patients meeting any criterion were classified as having heart failure.

### Diabetes Mellitus

Diabetes mellitus (DM_FLAG) was defined using three independent EHR signals:

1. Diagnosis codes: diabetes-related DBCs (e.g., 0335.222, 0318.902, 0313.223, 0316.7113, 0313.222, 0316.7114, 0313.221, 0305.2065, 0303.432), excluding gestational diabetes and diabetes insipidus.
2. Laboratory criteria: HbA1c ≥42 mmol/mol or plasma glucose ≥18 mmol/L, excluding non-blood specimens and point-of-care measurements.
3. Medication: use of any glucose-lowering therapy, including insulin, metformin, sulfonylureas, DPP-4 inhibitors, GLP-1 receptor agonists, SGLT2 inhibitors, or thiazolidinediones.

Patients were classified as diabetic if any criterion was present.

### Hypercholesterolemia

Hypercholesterolemia (HC_FLAG) was defined by:

- Diagnosis codes: DBCs explicitly indicating hypercholesterolemia or dyslipidemia.
- Laboratory criteria: LDL cholesterol ≥5.0 mmol/L or total cholesterol ≥8.0 mmol/L, thresholds chosen to identify severe or familial hypercholesterolemia.

Lipid-lowering therapy was not used as a defining criterion, given its widespread use for secondary prevention in cardiovascular patients.

### Ischemic Heart Disease

Ischemic heart disease (IHD_FLAG) was defined using a composite of diagnosis-based, procedure-based, and biomarker-based electronic health record signals, evaluated relative to each outpatient visit.

1. Diagnosis codes (DBC)
   Patients were flagged as having ischemic heart disease if a prior Diagnosis Treatment Combination (DBC) indicative of ischemic heart disease was present before the outpatient visit. Included DBCs covered stable angina pectoris, unstable angina, ST-elevation myocardial infarction (STEMI), non–ST-elevation myocardial infarction (NSTEMI), and other ischemic heart disease–related diagnoses. For each patient and outpatient visit, the most recent qualifying DBC preceding the trigger date was used.
2. Coronary procedures
   Ischemic heart disease was also identified based on a history of coronary diagnostic or interventional procedures prior to the outpatient visit, including coronary angiography (CAG) and percutaneous coronary intervention (PCI). Procedure codes corresponding to diagnostic angiography and coronary revascularization were included. The most recent qualifying procedure before the trigger date was considered.
3. Biomarker evidence of myocardial injury
   Evidence of myocardial injury was defined as a markedly elevated cardiac troponin T concentration (>500 ng/L), based on either the most recent value or the maximum recorded value prior to the outpatient visit.

Patients were classified as having ischemic heart disease (IHD_FLAG = 1) if any of the above criteria were met prior to the outpatient visit. This composite definition was designed to capture both chronic coronary artery disease and prior acute ischemic events, while minimizing reliance on a single data source.

### Chronic kidney disease

Chronic kidney disease (CKD) was defined based on the most recent estimated glomerular filtration rate (eGFR) available prior to the outpatient visit. CKD stage 3 or higher was defined as eGFR <60 mL/min/1.73 m² (CKD60_FLAG), and advanced CKD as eGFR <30 mL/min/1.73 m² (CKD30_FLAG). eGFR values were derived from routine laboratory measurements as recorded in the electronic medical record.

### Implantable cardiac devices (ICD and CRT-D)

Presence of an implantable cardiac device was defined based on procedure-related electronic health record signals indicating implantation, replacement, revision, or follow-up of a cardiac implantable electronic device prior to the outpatient visit.

Patients were classified as having an implantable cardioverter-defibrillator (ICD) if any ICD-related procedure was documented before the trigger date (ICD_FLAG = 1).

Patients were classified as having cardiac resynchronization therapy with defibrillator (CRT-D) if procedure codes indicated implantation, replacement, or follow-up of a CRT-D device prior to the outpatient visit (CRT_D_FLAG = 1).

# Appendix C. Sensitivity analysis evaluating stricter minimum follow-up duration requirements for apparently event-free outpatient visits

| **Scenario** | **N** | **Events (%)** | **AUROC** | **AUPRC** | **Brier** |
| --- | --- | --- | --- | --- | --- |
| ≥550-day follow-up for non-events (main analysis) | 199,961 | 33,627 (16.8%) | 0.769 ± 0.003 | 0.419 ± 0.006 | 0.118 |
| ≥730-day follow-up for non-events (sensitivity analysis) | 193,200 | 33,627 (17.4%) | 0.768 ± 0.003 | 0.427 ± 0.006 | 0.121 |

# Appendix D. Endpoint-specific sensitivity analyses

| **Endpoint** | **AUROC (SD)** | **AUPRC (SD)** | **Brier score** |
| --- | --- | --- | --- |
| 2-year composite | 0.769 (0.003) | 0.418 (0.006) | 0.118 |
| 2-year hospitalization only | 0.751 (0.004) | 0.312 (0.014) | 0.097 |
| 2-year mortality only | 0.873 (0.004) | 0.335 (0.013) | 0.045 |
| 1-year composite | 0.766 (0.002) | 0.309 (0.007) | 0.084 |
