# Supplementary material for: Cardiology hospital admission risk prediction: training, internal validation and technical implementation in the electronic health record
Source: Eur Heart J Digit Health. 2026 Jul 6;7(7):ztag109. doi: 10.1093/ehjdh/ztag109 (PMC13419068; doi:10.1093/ehjdh/ztag109)
Supplement: ztag109_Supplementary_Data [file ztag109_supplementary_data.zip › supplement_1 CODE-EHR_CHARP_Checklist.docx]

# Supplementary Appendix 1

## CODE-EHR Framework Checklist

Best practice checklist for reporting the use of structured electronic healthcare records in clinical research

Date of completion: [insert date]

Study name: Cardiology Hospital Admission Risk Prediction (CHARP): Training, Internal Validation and Technical Implementation in the Electronic Health Record

## 1. Dataset construction and linkage

Objective

To provide an understanding of how structured healthcare data were identified, constructed, and used in the study.

Minimum information provided

The dataset consisted of structured electronic health record (EHR) data from Amsterdam University Medical Centre (locations AMC and VUMC), extracted from Epic Systems and harmonized within curated Snowflake data views. Data domains included demographics, vital signs, laboratory results, medications, healthcare utilization, procedures, and imaging metadata. Each observation represented an outpatient cardiology visit (“trigger event”).

Missing data proportions for all major variables are reported in Table 1. Missingness was retained as observed, allowing the model to use absence of measurements as potentially informative. Follow-up completeness requirements were explicitly defined in the Methods section. Mortality data were obtained through linkage with municipal registries; linkage procedures follow institutional standards for pseudonymized patient identifiers.

Preferred information

A flow diagram describing dataset construction and preprocessing steps is provided in Appendix A.

Lead author acknowledgment

(3) Preferred standard met

## 2. Data fit for purpose

Objective

To ensure transparency regarding the origin, coding, and clinical context of structured healthcare data.

Minimum information provided

All data originated from routine clinical care within the Epic EHR environment. Data were recorded as part of standard clinical workflows and subsequently harmonized for research use. Coding systems included SNOMED CT for clinical concepts, LOINC for laboratory measurements, and ATC classification for medication exposure. Variable construction and feature derivation processes are described in detail in the Methods section and Appendix B.

Quality assurance consisted of standardized data harmonization across sites and manual verification of selected derived variables (e.g., left ventricular ejection fraction extraction). Potential sources of bias related to clinical workflow, selective testing, and missingness patterns are discussed in the Methods and Discussion sections.

Lead author acknowledgment

(3) Preferred standard met

## 3. Disease and outcome definitions

Objective

To fully describe disease phenotypes and outcome definitions to enable reproducibility.

Minimum information provided

Definitions for comorbidities, treatments, procedures, and outcomes were specified prior to statistical analysis. Detailed operational definitions and implementation logic for hypertension, heart failure, diabetes mellitus, hypercholesterolemia, ischemic heart disease, chronic kidney disease, and implantable cardiac devices are provided in Appendix B. Outcome definitions for unplanned cardiac hospitalization and all-cause mortality are described in the Methods section.

Phenotyping algorithms were based on combinations of diagnosis-treatment combinations (DBCs), laboratory thresholds, procedures, medication exposure, and device records. Definitions were developed based on institutional clinical practice and previously validated operational workflows.

Lead author acknowledgment

(2) Minimum standard met

## 4. Analysis

Objective

To enable independent assessment of analytical methods and reproducibility of findings.

Minimum information provided

Statistical and machine learning analyses were fully described in the Methods section. Risk prediction was performed using gradient-boosted decision trees (XGBoost), with hyperparameter optimization using Optuna. Internal validation employed patient-level GroupKFold cross-validation to prevent data leakage. Model performance was assessed using AUROC, AUPRC, calibration curves, and Brier score.

Explainability was evaluated using both model-level feature importance and trigger-level SHAP values. Generalizability was assessed through site-exclusive and patient-exclusive validation scenarios across two hospital locations.

Preferred information

The modeling framework, feature engineering strategy, and leakage-prevention procedures are described in detail, and code availability is stated in the Code Availability section.

Lead author acknowledgment

(3) Preferred standard met

## 5. Ethics and governance

Objective

To ensure transparency regarding ethical oversight, data security, and governance.

Minimum information provided

The study protocol was reviewed by the Medical Ethics Committee of Amsterdam UMC and classified as not subject to the Medical Research Involving Human Subjects Act (WMO). The requirement for informed consent was waived. All data were pseudonymized and processed in accordance with the General Data Protection Regulation (GDPR).

Data processing occurred within secure institutional infrastructure. Individual-level data cannot be publicly shared due to privacy and governance restrictions. Access to de-identified data and modeling code may be considered upon reasonable request and institutional approval.

Lead author acknowledgment

(2) Minimum standard met

## 6. Coding manual

DOI or website: Not applicable.

Operational definitions and coding logic are provided within Appendix B of the manuscript.

## 7. Comments

This study was conducted using routinely collected structured healthcare data within a single integrated healthcare system. The study design emphasizes transparency of variable definitions, leakage prevention, and implementation-oriented deployment within the electronic health record environment.

## 8. Summary declaration

All minimum standards met

Number of preferred standards met: 3 / 5
