# Supplementary material for: Cardiology hospital admission risk prediction: training, internal validation and technical implementation in the electronic health record
Source: Eur Heart J Digit Health. 2026 Jul 6;7(7):ztag109. doi: 10.1093/ehjdh/ztag109 (PMC13419068; doi:10.1093/ehjdh/ztag109)
Supplement: ztag109_Supplementary_Data [file ztag109_supplementary_data.zip › supplement_2 STROBE.docx]

# Supplementary Appendix 2

## STROBE Statement — Checklist of items that should be included in reports of cohort studies

Study: Cardiology Hospital Admission Risk Prediction (CHARP): Training, Internal Validation and Technical Implementation in the Electronic Health Record

| Item | Recommendation | Location in manuscript |
| --- | --- | --- |
| 1a | Indicate the study’s design with a commonly used term in the title or abstract | Title and Abstract (“retrospective cohort”, machine-learning model development and validation) |
| 1b | Provide in the abstract an informative and balanced summary of what was done and what was found | Abstract (Background, Methods, Results, Interpretation) |
| 2 | Explain the scientific background and rationale for the investigation | Introduction – Background and rationale |
| 3 | State specific objectives, including any prespecified hypotheses | Introduction – Aim of the Study |
| 4 | Present key elements of study design early in the paper | Methods – Dataset and Data Sources; Study design description |
| 5 | Describe the setting, locations, and relevant dates | Methods – Dataset and Data Sources (Amsterdam UMC AMC and VUMC, inclusion period January 2016–June 2025) |
| 6a | Give eligibility criteria and sources and methods of selection of participants. Describe methods of follow-up | Methods – Dataset and Data Sources; Outcome Definition; Leakage Prevention |
| 6b | For matched studies, give matching criteria | Not applicable (no matching performed) |
| 7 | Clearly define all outcomes, exposures, predictors, potential confounders, and effect modifiers | Methods – Outcome Definition; Feature Engineering; Appendix B |
| 8 | For each variable of interest, give sources of data and methods of assessment | Methods – Dataset and Data Sources; Feature Engineering; Appendix B |
| 9 | Describe efforts to address potential sources of bias | Methods – Leakage Prevention; Risk of Bias and Applicability (PROBAST+AI); Discussion – Limitations |
| 10 | Explain how the study size was arrived at | Methods – Dataset and Data Sources (all eligible outpatient visits included) |
| 11 | Explain how quantitative variables were handled in the analyses | Methods – Feature Engineering (LV, BLV, delta features, thresholds, derived variables) |
| 12a | Describe all statistical methods, including those used to control for confounding | Methods – Model Development and Evaluation |
| 12b | Describe methods used to examine subgroups and interactions | Methods – Evaluation; Results – Subgroup Analysis |
| 12c | Explain how missing data were addressed | Methods – Missing value handling |
| 12d | Explain how loss to follow-up was addressed | Methods – Leakage Prevention (minimum follow-up requirement) |
| 12e | Describe any sensitivity analyses | Methods – Leakage Prevention (follow-up sensitivity analysis) |
| 13a | Report numbers of individuals at each stage of study | Results – Study Characteristics; Table 1 |
| 13b | Give reasons for non-participation at each stage | Results – description of preprocessing exclusions |
| 13c | Consider use of a flow diagram | Appendix A – Workflow diagram |
| 14a | Give characteristics of study participants | Results – Baseline Characteristics; Table 1 |
| 14b | Indicate number of participants with missing data for each variable | Table 1 (Missing %) |
| 14c | Summarise follow-up time | Methods – Leakage Prevention; Outcome Definition |
| 15 | Report numbers of outcome events or summary measures over time | Results – Event Rates; Table 1 |
| 16a | Give unadjusted estimates and precision | Results – Model Performance (AUROC, AUPRC, Brier score) |
| 16b | Report category boundaries when continuous variables categorized | Methods – Feature Engineering; Appendix B |
| 16c | Translate estimates into absolute risk for meaningful time period | Results – Predicted 1-year and 2-year risk estimates |
| 17 | Report other analyses done (subgroups, sensitivity analyses) | Results – Subgroup Analysis; Generalizability and Cross-site Performance |
| 18 | Summarise key results with reference to study objectives | Discussion – opening paragraph |
| 19 | Discuss limitations and potential bias | Discussion – Limitations |
| 20 | Give cautious overall interpretation of results | Discussion – Interpretation and Clinical Implications |
| 21 | Discuss the generalisability (external validity) of the study results | Discussion – Generalizability and Cross-site Performance; Limitations |
| 22 | Give the source of funding and role of the funders | Funding section |
